# Fibrocytes in the fibrotic lung: altered phenotype detected by flow cytometry

**DOI:** 10.3389/fphar.2014.00141

**Published:** 2014-06-16

**Authors:** Charles Reese, Rebecca Lee, Michael Bonner, Beth Perry, Jonathan Heywood, Richard M. Silver, Elena Tourkina, Richard P. Visconti, Stanley Hoffman

**Affiliations:** ^1^Division of Rheumatology and Immunology, Department of Medicine, Medical University of South CarolinaCharleston, SC, USA; ^2^Department of Regenerative Medicine and Cell Biology, Medical University of South CarolinaCharleston, SC, USA

**Keywords:** collagen, scleroderma, CD45, CCR5, CXCR4, HSP47, CD44, caveolin-1

## Abstract

Fibrocytes are bone marrow hematopoietic-derived cells that also express a mesenchymal cell marker (commonly collagen I) and participate in fibrotic diseases of multiple organs. Given their origin, they or their precursors must be circulating cells before recruitment into target tissues. While most previous studies focused on circulating fibrocytes, here we focus on the fibrocyte phenotype in fibrotic tissue. The study's relevance to human disease is heightened by use of a model in which bleomycin is delivered systemically, recapitulating several features of human scleroderma including multi-organ fibrosis not observed when bleomycin is delivered directly into the lungs. Using flow cytometry, we find in the fibrotic lung a large population of CD45^high^ fibrocytes (called Region I) rarely found in vehicle-treated control mice. A second population of CD45+ fibrocytes (called Region II) is observed in both control and fibrotic lung. The level of CD45 in *circulating* fibrocytes is far lower than in either Region I or II lung fibrocytes. The chemokine receptors CXCR4 and CCR5 are expressed at higher levels in Region I than in Region II and are present at very low levels in all other lung cells including CD45+/collagen I- leucocytes. The collagen chaperone HSP47 is present at similar high levels in both Regions I and II, but at a higher level in fibrotic lung than in control lung. There is also a major population of HSP47^high^/CD45- cells in fibrotic lung not present in control lung. CD44 is present at higher levels in Region I than in Region II and at much lower levels in all other cells including CD45+/collagen I- leucocytes. When lung fibrosis is inhibited by restoring caveolin-1 activity using a caveolin-1 scaffolding domain peptide (CSD), a strong correlation is observed between fibrocyte number and fibrosis score. In summary, the distinctive phenotype of fibrotic lung fibrocytes suggests that fibrocyte differentiation occurs primarily within the target organ.

## Introduction

Fibrocytes are defined as bone marrow hematopoietic-derived cells that appear to be transitional in that they express leukocyte markers such as CD45 plus mesenchymal cell markers such as collagen I (Keeley et al., 2009; Pilling et al., 2009; Herzog and Bucala, 2010). Fibrocytes are present at high levels in the target tissue(s) in a variety of fibrotic diseases including scleroderma (systemic sclerosis, SSc) (Phillips et al., 2004; Falk, 2006; Haudek et al., 2006; Kisseleva et al., 2006; Wang et al., 2008; Mehrad et al., 2009; Niedermeier et al., 2009; Vakil et al., 2009; Mathai et al., 2010; Murray et al., 2011; Nikam et al., 2011). Given their origin in the bone marrow, it is clear that fibrocytes must traffic through the circulation to be recruited to target tissues through the action of chemokines on fibrocyte cell surface receptors. Once fibrocytes enter the target tissue, they participate in the progression of fibrosis by differentiating into fibroblasts and/or by secreting cytokines that activate resident fibroblasts.

Most previous studies on fibrocytes have been performed using human blood because of the ease in obtaining this material from control subjects and patients with a variety of different diseases. For example, a high level of circulating fibrocytes is an indicator of poor prognosis in patients with idiopathic pulmonary fibrosis (IPF) (Moeller et al., 2009). Very few studies have been performed on the quantification and phenotype of fibrocytes in target tissues such as the lung. Despite the focus in the literature on circulating fibrocytes, it appears that there are many more fibrocytes in the fibrotic lung than in the circulation (Phillips et al., 2004; Ishida et al., 2007). Moreover, little work has been done on the phenotypic characterization of lung fibrocytes beyond their expression of CD45, collagen I, and the chemokine receptor CXCR4. This is particularly noteworthy because while it has been pointed out that there appear to be too few circulating fibrocytes to account for the large number of fibrocytes in the fibrotic lung (Phillips et al., 2004), the possibility has not been examined that this may occur because fibrocyte differentiation is occurring primarily in the lungs (or in other fibrotic target organs).

Two recent technical advances have been instrumental in allowing us to perform studies on the quantification and phenotyping of lung fibrocytes. The most common approach to inducing lung fibrosis in mice is to deliver bleomycin directly into the lungs, usually by intratracheal administration. However, we recently observed that systemic administration of bleomycin using osmotic minipumps produces a disease that more closely resembles human SSc than does direct administration of bleomycin into the lungs (Lee et al., 2014). Features that are common to the Pump Model and SSc, but not to the Direct Model, include: (1) Fibrosis is limited to the subpleural portion of the lung; (2) Inflammation is relatively limited; (3) Hypertrophic type II alveolar epithelial cells are present at high levels; and (4) Lung fibrosis is accompanied by dermal fibrosis and fibrosis of other internal organs. A second issue that we have addressed is that previous studies on fibrocytes have shown that it is difficult to determine clearly by flow cytometry which CD45+ cells are also collagen I+. This distinction is difficult because, in reality, collagen I expression levels are not categorical (i.e., negative or positive), but rather a continuum of collagen I expression levels exists. In addition, because most anti-collagen I antibodies used in flow cytometry are rabbit polyclonal antibodies, the isotype control is non-immune rabbit IgG which can vary in degree of non-specific labeling, possibly due to the intrinsic heterogeneity of the preparations. We have addressed this problem by making a polyclonal antibody against a polypeptide from the human collagen Iα1 C-terminal propeptide. Using this antibody, we can ensure that we are labeling cells that express collagen Iα1 rather than cells that bind mature collagen (lacking this propeptide) and we can distinguish specific antibody binding from non-specific binding by using the immunogenic peptide in competition experiments.

In the current study, a comparison by flow cytometry of the number and phenotype of lung fibrocytes in mice treated with bleomycin or saline vehicle utilizing our Pump Model has allowed us to make several striking, novel observations regarding the levels of CD45, collagen I, CD14, CD34, CXCR4, CCR5, HSP47, and CD44 in lung fibrocytes and to demonstrate that there is a high correlation between fibrocyte number and fibrosis severity in fibrotic lung tissue. These observations suggest that fibrocyte differentiation occurs primarily in the lung and that treatments that target fibrocyte recruitment and differentiation [in particular administration of the caveolin-1 scaffolding domain peptide (CSD)] are likely to be effective treatments for fibrotic diseases of the lung and other organs.

## Materials and methods

### Bleomycin treatment of mice and harvesting of tissue

The following procedures were approved by the MUSC Institutional Animal Care & Use Committee. Ten-week old, male CD1 mice (Charles River Laboratories, Boston, MA) were maintained under pathogen-free conditions. Osmotic minipumps (ALZET 1007D; DURECT Corporation, Cupertino, CA) containing either 100 μl saline vehicle or bleomycin (100 U/kg) designed to deliver their contents at 0.5 μ l/h for 7 days were implanted under isofluorane anesthesia under the loose skin on the back of the mice slightly caudal to the scapulae. Pumps were removed on day 10–12.

In the experiment shown in Figure 10, at the same time that the pump containing bleomycin or saline was removed, a second pump (ALZET 2002, designed to deliver its contents of 200 μl at 0.5 μl/h for 14 days) was inserted containing CSD peptide (5 mg per ml in 10% DMSO in water) or vehicle.

Mice were sacrificed on the indicated day. Under deep anesthesia, the rib cage was opened to expose the lungs. Mice were systemically perfused via the left ventricle with PBS. The left lung was then tied off and removed for flow cytometry experiments. The remaining right lung was further perfused with buffered zinc formalin fixative (Z-Fix, Anatech, Battle Creek, MI). Fixed lung tissue was then removed and embedded in paraffin. Sections (4 μm) were stained with hematoxylin and eosin (H&E) or Masson's Trichrome.

### Flow cytometry

Lung tissue prepared as described above were washed in cold DMEM and minced into small pieces using fine surgical scissors. The tissue from each mouse was resuspended in 5 ml of collagenase (1 mg/ml, Roche #10103578001) and shaken at low speed for 45 min at 37°C. Digestion was stopped by addition of 10 ml of DMEM/10% FBS. The sample was passed through a 40 μm cell strainer to prepare a monodisperse cell suspension. Cells were collected by centrifugation (1200 rpm, 10 min, 4°C, Beckman GS-6R centrifuge) and the cell pellets resuspended in 1 ml ACK lysing buffer (LONZA #10-548E) for 5 min to remove red blood cells. Lung cells, approximately ten million per mouse, were then collected by centrifugation, washed with PBS, collected, and fixed and permeabilized for 20 min at 4°C in 1 ml Cytofix/Cytoperm (BD #554722). Finally, cells were washed in FACS buffer (BD Perm/Wash Buffer 554723) and resuspended at 4 × 10^6^ per ml in FACS buffer/Fc Block (BD, 1 μg per 1×10^6^ cells).

Antibodies used in immunolabeling are summarized in Table 1. All incubations with antibody were 30 min at 4°C on a rocker at low speed, all washes were done with FACS buffer, all resuspensions were with FACS buffer/Fc Block. Prior to use, the antibodies designated Pro or Telo were preincubated with specific or non-specific peptide (30 min, RT, 10 μg per ml IgG in FACS buffer plus 20 μg per ml peptide). (Peptides had been reduced and alkylated as described below). The antibody/peptide mixture (50 μl) was then incubated with 0.25 ml lung cells. Following washing, the cells were second incubated with anti-rabbit secondary antibody. Following washing, the cells were simultaneously third incubated with directly-labeled anti-CD45 and with directly-labeled antibodies against either CD14, CD34, Rockland anti-collagen I, CXCR4, CCR5, HSP47, or CD44. Following washing, the immunolabeled cell suspension was analyzed by FACS using a MoFlo Astrios (Beckman Coulter). At least 20,000 events were recorded per sample.

**Table 1:** **Antibodies used in flow cytometry experiments**.

**Specificity (description)**	**Label**	**Product #**	**Concentration**
CD45 (rat anti-mouse, clone 30-F11)	PE-Cy7	BD 552848	0.50 μg/ml
Collagen Iα1 C-terminal propeptide “Pro” (rabbit anti-human, polyclonal)	None		1.67 μg/ml
CD14 (rat anti-mouse, clone rmC5-3)	APC	BD 560634	0.75 μg/ml
CD34 (rat anti-mouse, clone RAM34)	AF647	BD 560230	1.50 μg/ml
Collagen I N-terminal propeptide (rat anti-human, clone M-58)	None	EMDMillipore MAB1912	1.00 μg/ml
Collagen I C-terminal propeptide (mouse anti-human, clone PCIDG10)	None	EMDMillipore MAB1913	1.00 μg/ml
Collagen I (rabbit anti-human/bovine, polyclonal)	AF647^*^	Rockland 600-401-103	1.00 μg/ml
CXCR4 (rat anti-mouse, clone 2B11)	APC	eBioscience 17-9991-82	1.00 μg/ml
CCR5 (Armenian Hamster anti-mouse, clone HM-CCR5)	APC	BioLegend 107012	1.50 μg/ml
HSP47 (mouse anti-rat, clone M16.10A1)	AF647^*^	Enzo ADI-SPA-70	1.00 μg/ml
CD44 (rat anti-mouse, clone IM7)	APC	BD 559250	0.50 μg/ml
CD16/CD32 “Fc Block” (rat anti-mouse, clone 2.4G2)	None	BD 553142	1 μg/1 × 10^6^ cells
Goat anti-rabbit secondary	AF488	Life technologies A11034	2.00 μg/ml
Goat anti-mouse secondary	AF647	Life technologies A21235	2.00 μg/ml
Goat anti-rat secondary	AF647	Life technologies A21247	2.00 μg/ml

* *Labeled using an Alexa Fluor 647 Protein Labeling Kit (Invitrogen, Carlsbad, CA, A-20173) as described by the manufacturer. AF, Alexa Fluor*.

In experiments which involved immunolabeling CD45, Pro, and MAB1912 or MAB1913, because MAB1912 and MAB1913 were not available directly labeled and could not be directly labeled in our laboratory, it was necessary to simultaneously first label with Pro and MAB1912 or MAB1913, then to simultaneously second label with appropriate secondary antibodies. As above, in a third incubation the cells received directly-labeled anti-CD45.

In experiments on circulating fibrocytes, blood (typically about 1 ml) was collected by heart stick into a tube already containing heparin (500 USP units) as anti-coagulant. PBMC were isolated by routine methods and immunolabeled as described above.

### Collagen peptide antibodies

19-amino acid peptides from the human collagen Iα1 C-terminal propeptide and C-terminal telopeptide were synthesized with an additional Cys residue at the N-terminus. This residue was used to link the peptides to KLH for immunization of rabbits and to Sulfolink beads (Thermo Fisher Scientific Inc., Rockford, IL). Total IgG was purified from rabbit serum. Specific IgG was then affinity purified from total IgG using the immunogenic peptides attached to Sulfolink beads. After elution, affinity-purified IgG was spin-concentrated and desalted into PBS.

### Reduction/alkylation of peptides

10 mg of the immunogenic peptide for Pro and for Telo was dissolved in 1 ml TE (50 mM Tris, pH 8.5/5 mM EDTA). Next, 50 μl of 1 M DTT in TE was added and incubated 30 min, 37°C. Following clarification of the solution, the peptides were alkylated by addition of 225 μl of 0.67 M iodoacetimide in TE and incubation for 30 min, 37°C. Finally the peptides were desalted by gel filtration in PBS on a 10-ml Sephadex G-15 column. Peak fractions were pooled and mixed with an equal volume of 100% glycerol for storage in solution at −20°C.

### Fibrosis scores

Fibrosis was quantified by light microscopic evaluation of tissue sections. Briefly, for each mouse a Masson's Trichrome-stained slide and an H & E-stained slide were scored blind using an arbitrary scale in which normal morphology = 0; almost normal = 1; slightly altered morphology = 2; moderately altered morphology = 3; and severely altered morphology = 4. After the blinding code was broken, the final score for each mouse was determined by adding its Masson's Trichrome score to its H & E score.

### EGFP mice

Ten to 14-week-old male C57BL6/Ly5.1 mice were prepared for bone marrow transplantation using a single dose (9.0 Gy) of total body irradiation. Ten to 12-week-old female EGFP/Ly5.2 mice were used as donors (Visconti et al., 2006). Briefly, donor mice were humanely sacrificed by CO_2_ inhalation. Bone marrow cells were flushed from femurs and tibiae and washed in Ca^++^- and Mg^++^-free phosphate-buffered saline containing 0.1% BSA. A monodisperse suspension was prepared by gentle trituration and filtering through a 40-μ m filter. Mononuclear cells were isolated by density gradient centrifugation using Lympholyte-M (Cedarlane Labs, Burlingame, NC). 2.0×10^5^ of these EGFP+ donor bone marrow cells were transplanted into the irradiated recipients by tail vein injection. As previously described (Visconti et al., 2006), peripheral blood chimerism was assayed at 30 days post-transplantation and multi-lineage hematopoietic reconstitution was assayed at 60 days. Mice exhibiting high levels of reconstitution were used in subsequent studies.

### Statistical analyses

All numerical data are expressed as average ± standard error of the mean (s.e.m.) and were analyzed using Student's *t*-test to evaluate statistical significance. In all figures, statistical significance is expressed as ^*^p < 0.05, ^**^p < 0.01, and ^***^p < 0.002.

## Results

### Identification of CD45^high^/pro+ cells found only in bleomycin lung

**Figure 1:**
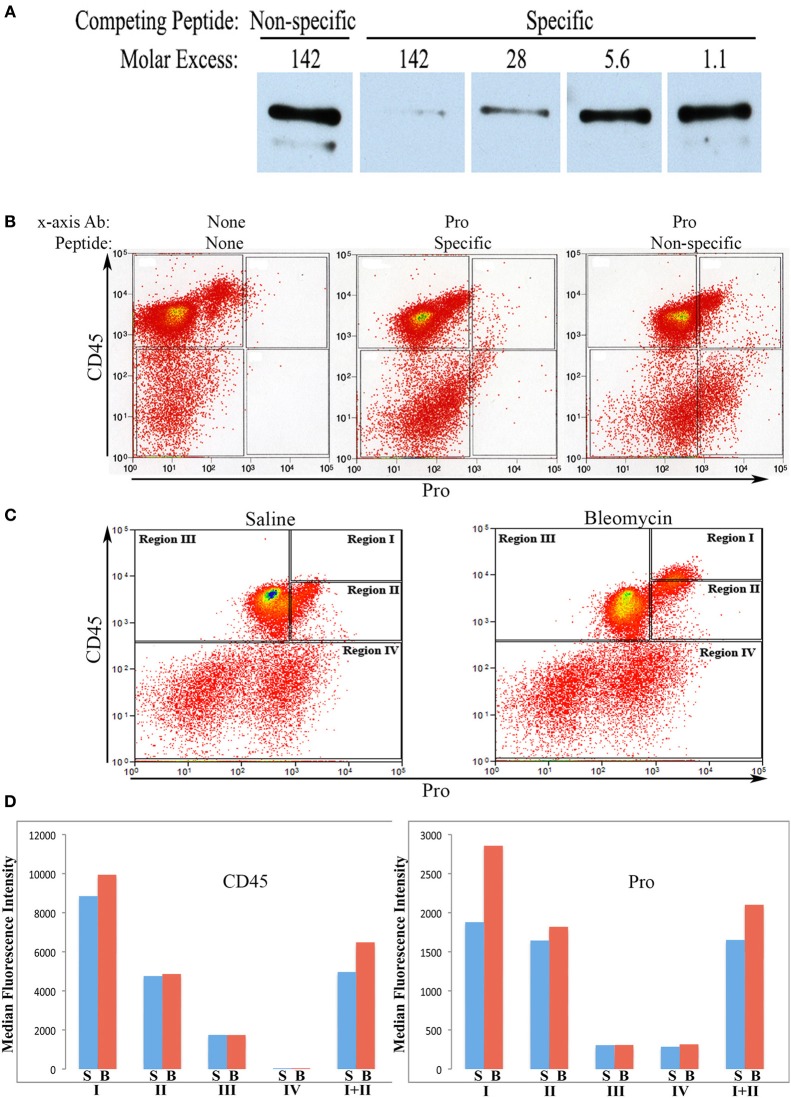
**Characterization of Pro by Western Blot and use in flow cytometry. (A)**Human lung fibroblast culture supernatant was Western blotted using Pro (0.2 μg/ml) competed with Non-specific peptide (immunogenic peptide for Telo, 0.4 μg/ml = 142-fold molar excess), with Specific peptide (immunogenic peptide for Pro, 0.4 μg/ml = 142-fold molar excess), or with serial 5-fold dilutions of Specific peptide. The prominent polypeptide detected is full-length 180 kD procollagen Iα1. The minor, slightly smaller polypeptide lacks the N-terminal propeptide. **(B)** Total lung cells from a bleomycin-treated mouse sacrificed 28 days after initiation of treatment were immunolabeled for CD45 and, as indicated, with no additional antibody, with Pro competed with specific peptide (142-fold molar excess), and with Pro competed with non-specific peptide (142-fold molar excess). Scatter plots are presented with CD45 fluorescence intensity on the y-axis and Pro (or unlabeled) fluorescence intensity on the x -axis. **(C)** Total lung cells from a saline-treated mouse and a bleomycin-treated mouse sacrificed 28 days after initiation of treatment were immunolabeled for CD45 and Pro. Scatter plots are presented with CD45 fluorescence intensity on the y-axis and Pro fluorescence intensity on the x-axis. The zones named Regions I–IV are shown and are used throughout this study. **(D)** CD45 or Pro levels in Regions I–IV, and I + II are shown in terms of the average ± s.e.m. of the median fluorescence intensity of each of 18 saline and 26 bleomycin mice sacrificed 28 days after initiation of treatment. S, saline; B, bleomycin. Error bars are not shown and the *t*-test was not applied to these data because CD45 and Pro fluorescence intensity were the criteria used to define Regions I–IV.

Rabbit polyclonal antibodies against the human collagen Iα1 C-terminal propeptide and C-terminal telopeptide were prepared as described in the Methods. We refer to the propeptide antibody as Pro and to the telopeptide antibody as Telo. In this paper we only show flow cytometry data obtained with Pro, although similar data were obtained with Telo. To characterize Pro, we first demonstrated by Western blotting that its activity can be almost completely blocked in a dose-dependent manner by its specific immunogenic peptide, but not by the non-specific immunogenic peptide for Telo (Figure 1A).

**Figure 2:**
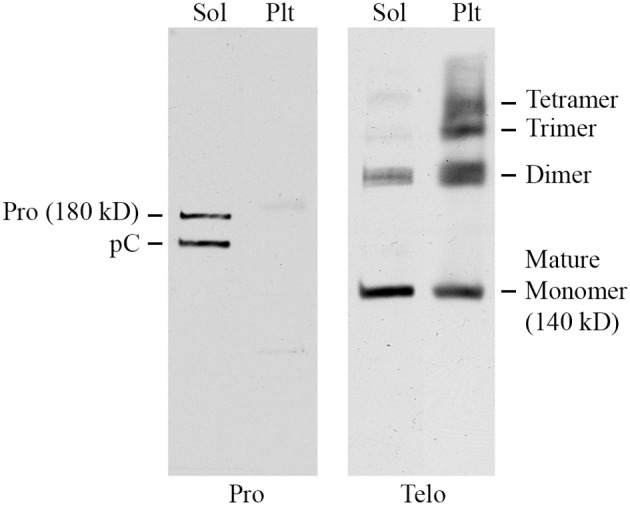
**Specificity of Pro and Telo by Western Blotting of tissue extracts**. A soluble tissue extract of shaved mouse skin was prepared using detergent-free neutral buffer as previously described for lung tissue (Lee et al., 2014). Following centrifugation, the pellet was re-extracted by boiling for 3 min in SDS-PAGE sample buffer. Aliquots of the soluble extract (Sol) and the pellet extract (Plt) representing equal amounts of tissue were then resolved by SDS-PAGE and Western blotted using Pro and Telo as indicated. The identity of the various forms of collagen I recognized by these antibodies is described in the text and indicated on this figure.

To further characterize Pro and Telo, the ability of these antibodies to detect collagen I on Western blots of mouse tissue extracts was determined. Skin was used for these experiments because it contains a higher level of collagen I than does lung. Skin was homogenized sequentially with neutral buffer (to extract soluble collagen I that had not yet been incorporated into the extracellular matrix) followed by SDS-PAGE sample buffer extraction of the pellet (to extract collagen I that had begun to be cross-linked into the extracellular matrix, but was not fully insoluble). As expected (Figure 2), Pro detected full-length 180 kD procollagen I and the slightly smaller pC (procollagen I lacking the N-terminal propeptide) in the soluble fraction but did not recognize any form of collagen I present in the pellet fraction because propeptides are removed from collagen I prior to its incorporation into the extracellular matrix. In sharp contrast (Figure 2) and as expected, Telo recognized primarily 140 kD mature (i.e., lacking propeptides) collagen I monomers and some dimers in the soluble fraction but recognized a higher level of collagen I in the pellet fraction, most of which was covalently crosslinked into dimers, trimers, and tetramers. While 180 kD procollagen I and pC are not shown to be detected by Telo in Figure 2, they are observed on longer exposures of the same Western blot. In summary, Figure 2 demonstrates that procollagen I is almost completely in the soluble fraction and that the great majority of collagen I in tissue is the mature molecule or multimers of the mature molecule and is present in the pellet fraction. Figure 2 further demonstrates that Pro and Telo have the expected, distinct specificities and do not appear to recognize any proteins other than forms of collagen I.

We next performed a competition experiment by flow cytometry. Total lung cells from a mouse treated with bleomycin using the Pump Model (Lee et al., 2014) and sacrificed at day 28 were double-labeled for CD45 and Pro with competition with either the Pro or Telo immunogenic peptides (Figure 1B). Specific competition showed no CD45+ cells with a fluorescence intensity >9×10^2^ although with non-specific competition there was a large population of CD45+/Pro+ cells using this cutoff. Experiments with cells immunolabeled only for CD45 confirmed that 9×10^2^ is an appropriate cutoff for specific labeling. Having established criteria for Pro+ labeling, we compared total lung cells from Pump Model mice treated with bleomycin or with saline vehicle (Figure 1C). Examination of data from 18 saline-treated and 26 bleomycin-treated mice has allowed us to divide CD45/Pro scatter plots into four Regions: Region I, CD45^high^/Pro+ (CD45^high^ = fluorescence intensity >8× 10^3^); Region II, CD45+/Pro+ (CD45+ = fluorescence intensity >5×10^2^); Region III, CD45+/Pro-; and Region IV, CD45-. Strikingly, a significant number of cells are present in Region I only in mice treated with bleomycin. The median fluorescence intensity for CD45 and Pro in Regions I–IV from saline and bleomycin mice (Figure 1D) is in accord with the position of these Regions in the scatter plots in Figure 1C.

**Figure 3:**
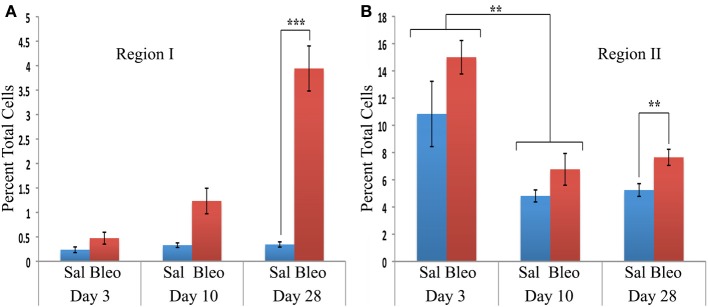
**Time course of cell numbers in Regions I and II**. The percent of total lung cells in Region I **(A)** or II **(B)** in saline- or bleomycin-treated mice 3, 10, or 28 days after initiation of treatment is shown. Sal, saline; Bleo, bleomycin. These data represent the average ± s.e.m. for the following number of mice in each category: 3-day saline, 3; 3-day bleomycin, 3; 10-day saline, 3; 10-day bleomycin, 3; 28-day saline, 18; 28-day bleomycin, 26. Because there was no difference between the percent of cells in Region II in saline and bleomycin lung at day 3 and at day 10, saline and bleomycin data were pooled before determining the statistical significance in the decrease in percent of cells in Region II between day 3 and 10. ^**^p < 0.01, ^***^p < 0.002.

When the time course of accumulation of cells in Regions I and II was examined (Figure 3), in Region I the number of cells in mice receiving Saline vehicle was always low while the number in bleomycin-treated mice rose dramatically from day 3 to days 10–28. Interestingly, although the percentage of cells in Region II in naïve mice was no more than 4% (not shown), this number increased to over 10% in both saline and bleomycin-treated mice 3 days after pump implantation before returning to almost baseline level at 10 days, indicating that the increase at 3 days is due to pump implantation and not the contents of the pump. At 28 days, the level in saline mice remained at the 10-day level, while the level in bleomycin mice increased slightly and was statistically significantly different from the level in saline mice. In summary, these studies show that there is always a population of CD45+Pro+ (Region II) cells in mouse lung, but there is a rather large population of CD45^high^/Pro+ (Region I) cells that are only present in bleomycin-treated mice and that take time to accumulate.

To confirm the identity of Region I and Region II cells as bone marrow-derived cells, similar experiments were performed in mice reconstituted with bone marrow expressing EGFP (not shown). In both saline- and bleomycin-treated mice, consistent with their expression of the hematopoietic cell marker CD45, cells in Regions I–III were 100% EGFP+, while cells in Region IV were only about 10% EGFP+.

### CD14 and CD34

**Figure 4:**
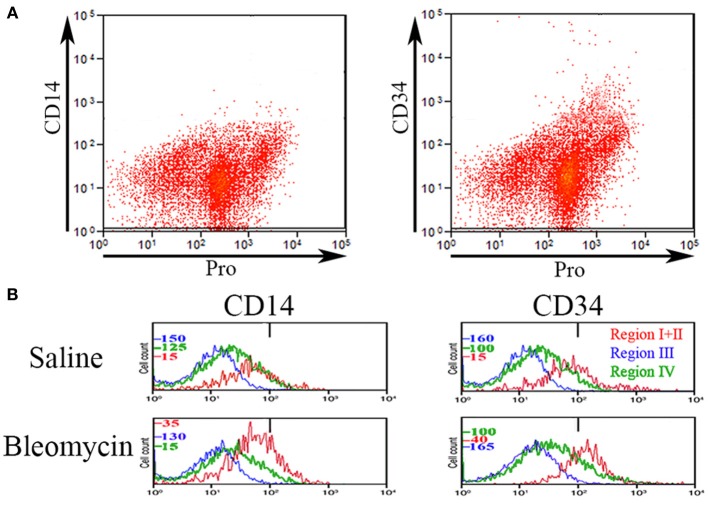
**CD14 and CD34 in Regions I–IV. (A)** Total lung cells from a bleomycin-treated mouse sacrificed 28 days after initiation of treatment were immunolabeled for Pro and either CD14 or CD34. Scatter plots of fluorescence intensity are presented with CD14 or CD34 on the y-axis and Pro on the x-axis. Note that unlike CD45 (see Figure 1), neither CD14 nor CD34 had a clearcut bimodal distribution of fluorescence intensity. **(B)** Total lung cells from a saline- and a bleomycin-treated mouse sacrificed 28 days after initiation of treatment were immunolabeled for CD45, Pro, and either CD14 or CD34. Cells from the indicated Regions (the indication under CD34 applies to CD14 as well) from CD45-Pro scatter plots were selected and the fluorescence intensity of CD14 or CD34 in these cells plotted vs. cell count. The bar at 1×10^2^ marks the maximum value of fluorescence intensity (99th percentile) detected in unlabeled cells. The different cell count scales for each Region are indicated. Similar results were observed in three independent experiments.

Various authors have considered the status of CD14 and CD34 expression to be important in the characterization of circulating fibrocytes. Therefore, we performed experiments in which total lung cells from saline- and bleomycin-treated mice sacrificed 28 days after treatment were immunolabeled for CD45, Pro, and either CD14 or CD34 (Figure 4). Unlike CD45 in which a bimodal immunolabeling distribution is obvious in both saline and bleomycin lung (Figure 1C), neither CD14 nor CD34 show a bimodal distribution (Figure 4A). CD14 and CD34 levels in cells from Regions I–IV of saline- and bleomycin-treated mice were also compared (Figure 4B). Data for Regions I and II were pooled because there was no difference between these regions. For CD14, essentially no cells in Region III were positive, while a similar percentage of cells in both saline- and bleomycin-treated mice were positive in Regions I + II (17.5%). For CD34, again essentially no cells in Region III were positive. In contrast to CD14, the percentage of CD34+ cells was higher in bleomycin lung than in saline lung in Regions I + II (69 vs. 40%).

### Comparison of pro with other collagen I antibodies

**Figure 5:**
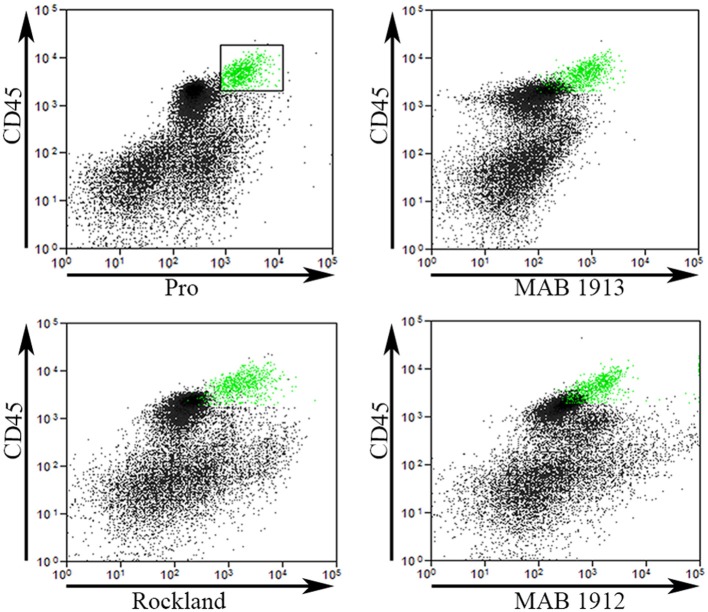
**Comparison of Pro to other collagen antibodies**. Total lung cells from a bleomycin-treated mouse sacrificed 28 days after initiation of treatment were immunolabeled for CD45, Pro, and one of MAB1913, MAB1912, or Rockland. In the upper left panel, a scatter plot is shown with CD45 on the y-axis, Pro-on the x-axis, and a box containing Regions I and II with the cells inside the box selected and marked in green. In the other three panels, scatter plots are shown with CD45 on the y-axis and, as indicated, MAB1913, MAB1912, or Rockland on the x-axis. The same cells marked green due to their position on CD45-Pro scatter plots are green on these plots. Note that the green cells are in the same position on all panels indicating that the same cells are recognized by Pro, MAB1913, MAB1912, and Rockland. In contrast to the similarity in all four panels among CD45+ cells, note that, as described in the text, differences are seen in the position of CD45- cells. Similar results were observed in three independent experiments.

To validate our Pro antibody and to compare cells recognized by various collagen I antibodies, we performed experiments in which total lung cells from bleomycin-treated mice sacrificed 28 days after treatment were immunolabeled for CD45, Pro, and one of EMD Millipore MAB1913 (Mouse monoclonal anti-human collagen Iα1 C-terminal propeptide), EMD Millipore MAB1912 (Rat monoclonal anti-human collagen Iα1 N-terminal propeptide), or Rockland Rabbit polyclonal anti-human and bovine collagen I (this is the most frequently used antibody for detecting fibrocytes by flow cytometry, particularly in experiments using mouse samples). The indicated cells from Regions I and II in a scatter plot of CD45 vs. Pro were marked green and then these same cells were localized in scatter plots of CD45 vs. MAB1913, MAB1912, and Rockland (Figure 5). In every case the green marked cells exhibited a similar distribution on the scatter plot indicating that this population of CD45+ cells is also recognized by each of these four anti-collagen antibodies. In contrast, these four antibodies differed in their ability to recognize CD45- cells. The most Pro+ CD45- cells had a similar fluorescence intensity to the green marked cells, the most MAB1913+ CD45- cells had less fluorescence than the green marked cells, the most Rockland+ CD45- cells had more fluorescence than the green marked cells, and the most MAB1912+ CD45- cells had far more fluorescence than the green marked cells (Figure 5). Whether these differences result from the recognized epitopes being differentially expressed or being differentially available due to distinctive patterns of subcellular localization, masking, or presence on different molecular forms of collagen I (e.g., fragments or fibrils) is an open question. It is also noteworthy that we have obtained positive results using MAB1912 and MAB1913 on mouse samples by flow cytometry and by immunohistochemistry (submitted for publication) because these antibodies were previously thought not to recognize mouse collagen I.

### Chemokine receptors CXCR4 and CCR5

**Figure 6:**
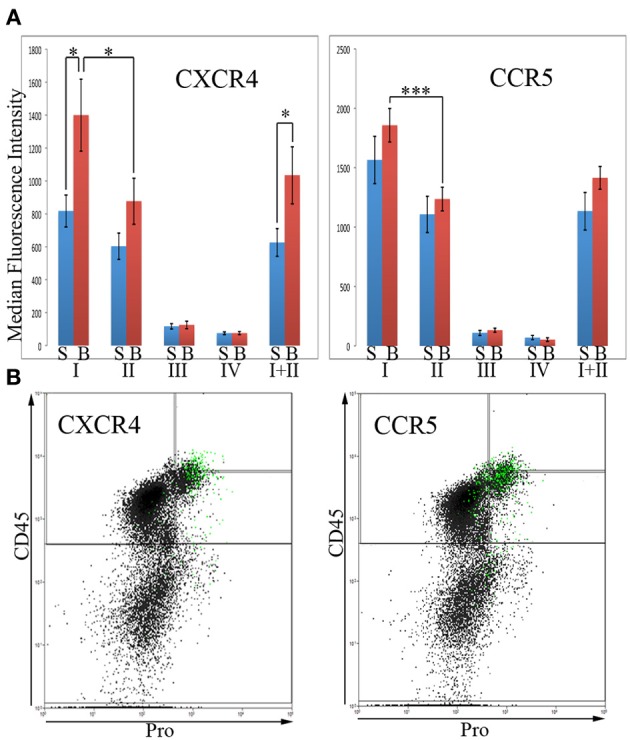
**Chemokines CXCR4 and CCR5 in Regions I–IV**. **(A)**Total lung cells from saline- and bleomycin-treated mice sacrificed 28 days after initiation of treatment were immunolabeled for CD45, Pro, and either CXCR4 or CCR5. CXCR4 or CCR5 levels in Regions I–IV, and I + II are shown in terms of the average ± s.e.m. of the median fluorescence intensity of each of the following number of mice: CXCR4, 15 saline and 18 bleomycin; CCR5, 9 saline and 12 bleomycin. S, saline; B, bleomycin. **(B)** The 2% of the total lung cells with the highest levels of CXCR4 or CCR5 were selected and marked green. Scatter plots of CD45 on the y-axis vs. Pro on the x-axis showing these selected, green marked cells are presented. As noted in the text, the position of the green-marked cells is different for CXCR4 and CCR5. Similar results were observed in three independent experiments. ^*^p < 0.05, ^***^p < 0.002.

In order for fibrocytes to play a role in fibrosis, it is necessary for them or their progenitors to be recruited into target tissues. In this process chemokines secreted by cells in target tissues promote recruitment into the tissue by binding to specific receptors on circulating cells. Two of the most prominent of these chemokine receptors are CXCR4 and CCR5. In these experiments, total lung cells from saline- and bleomycin-treated mice sacrificed 28 days after treatment were immunolabeled for CD45, Pro, and either CXCR4 or CCR5. For both CXCR4 and CCR5, the level of fluorescence intensity was high in Regions I and II, while Regions III and IV were unlabeled or at most barely positive (Figure 6A). For example, for both CXCR4 and CCR5, the median fluorescence intensity in bleomycin Region I was > 10-fold higher than in bleomycin Regions III or IV. For both CXCR4 and CCR5, there was a significant difference in the fluorescence intensity between bleomycin Region I and bleomycin Region II. While the fluorescence intensity level for CXCR4 is significantly higher in bleomycin Regions I and I + II compared respectively to Saline Regions I and I + II, it is not for CCR5. It is also quite noteworthy that the cells that have the highest levels of CXCR4 are not the same cells that have the highest levels of CCR5 (Figure 6B). The green marked cells with the highest level of CXCR4 are skewed toward the highest levels of CD45 and Pro, while the green marked cells with the highest level of CCR5 are skewed toward the arbitrary border between Region III and Regions I and II and in many cases are slightly across the border.

### Collagen chaperone HSP47

**Figure 7:**
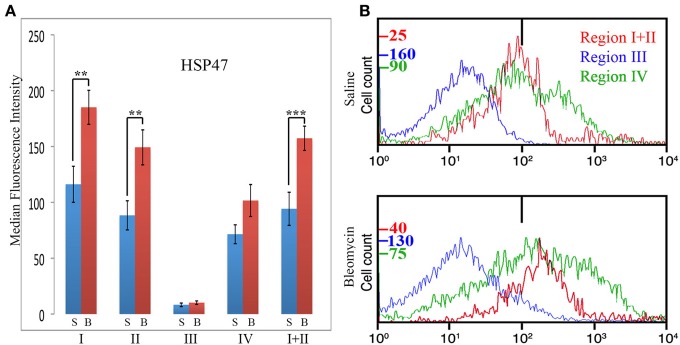
**Collagen Chaperone HSP47 in Regions I–IV. (A)** Total lung cells from saline- and bleomycin-treated mice sacrificed 28 days after initiation of treatment were immunolabeled for CD45, Pro, and HSP47. HSP47 levels in Regions I–IV, and I + II are shown in terms of the average ± s.e.m. of the median fluorescence intensity of each of 11 saline and 17 bleomycin mice. S, saline; B, bleomycin. **(B)** In some of the experiments that make up **(A)**, cells from the indicated Regions from CD45-Pro scatter plots were selected and the fluorescence intensity of HSP47 in these cells plotted vs. cell count. The bar at 1×10^2^ marks the maximum value of fluorescence intensity (99th percentile) detected in unlabeled cells. The different cell count scales for each Region are indicated. Note the large number of cells in bleomycin Region IV with a fluorescence intensity >1× 10^3^, a level not observed in any other region. Similar results were obtained in four independent experiments. ^**^p < 0.01, ^***^p < 0.002.

HSP47 is upregulated in a high percentage of the cells in fibrotic mouse and human lung tissue (Lee et al., 2014) and thus serves as a cell-associated, surrogate marker for collagen expression. In these experiments, total lung cells from saline- and bleomycin-treated mice sacrificed 28 days after treatment were immunolabeled for CD45, Pro, and HSP47 (Figure 7). Unlike CXCR4 and CCR5, only Region III showed little, if any, HSP47 immunolabeling. Regions I, II, I + II, and IV all showed a higher level of immunolabeling in bleomycin-treated mice than in saline-treated mice (Figure 7A). In particular, a major subpopulation of bleomycin Region IV (i.e., CD45- cells) with a fluorescence intensity >1×10^3^ (Figure 7B) is observed that is not present in bleomycin Region I + II or in saline Region IV. Cells with this high fluorescence intensity represent 4.5% of the cells in bleomycin Region IV, but only 1.5% of the cells in saline Region IV (*p* = 0.03). Also unlike CXCR4 and CCR5, there was not a statistically significant difference between bleomycin Region I and bleomycin Region II.

### CD44

**Figure 8:**
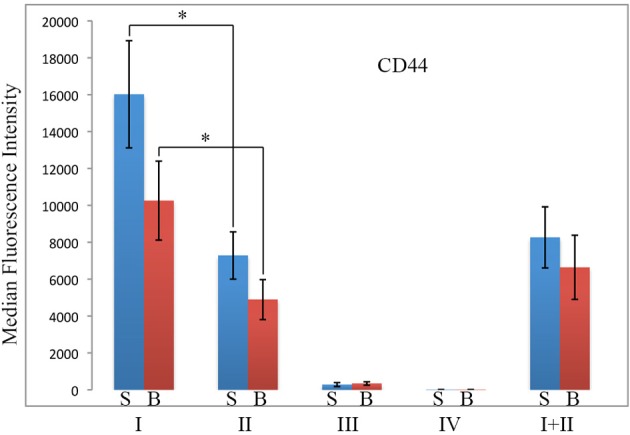
**CD44 in Regions I–IV**. Total lung cells from saline- and bleomycin-treated mice sacrificed 28 days after initiation of treatment were immunolabeled for CD45, Pro, and CD44. CD44 levels in Regions I–IV, and I + II are shown in terms of the average ± s.e.m. of the median fluorescence intensity of each of 9 saline and 11 bleomycin mice. S, saline; B, bleomycin. ^*^p < 0.05.

Because CD44 is considered to be an important functional marker on mesenchymal stem cells, we determined whether CD44 might also be present on fibrocytes. In these experiments, total lung cells from saline- and bleomycin-treated mice sacrificed 28 days after initiation of treatment were immunolabeled for CD45, Pro, and CD44 (Figure 8). While no statistically significant difference was observed for any Region between saline- and bleomycin-treated mice, huge differences were observed between Regions. CD44 immunolabeling was only at the unlabeled background level in Region IV. The level of fluorescence intensity was then about 20-fold higher in Region III, 400-fold higher in Region II, and 800-fold higher in Region I. The difference between Regions I and II was statistically significant, especially when the data from saline- and bleomycin-treated mice was pooled (*p* < 0.002). In summary, the level of CD44 in both Regions I and II is very high and is likely to be functionally important in the large population of Region I cells found in bleomycin lung.

### Blood fibrocytes

**Figure 9:**
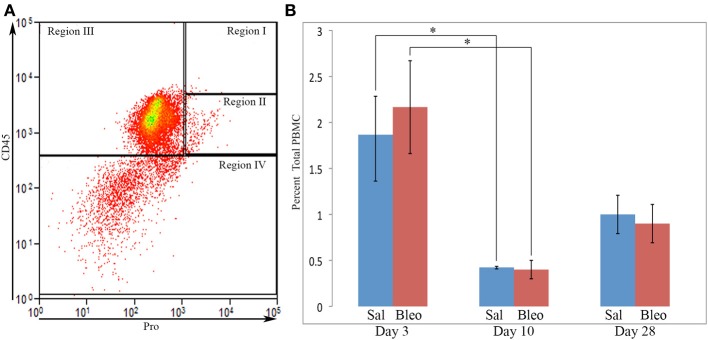
**Circulating fibrocytes. (A)** PBMC from a bleomycin-treated mouse sacrificed 28 days after initiation of treatment were immunolabeled for CD45 and Pro. A scatter plot is presented with CD45 on the y-axis and Pro on the x-axis showing almost no cells in Region I, few cells in Region II, and that, even among the few cells present in Region II, almost none were in the upper half of Region II where almost all lung cells in Region II are located. This distribution of cells was observed in all PBMC examined from saline- and bleomycin-treated mice sacrificed 3, 10, or 28 days after the initiation of treatment. **(B)** Percent of PBMC in Region I + II in saline- or bleomycin-treated mice 3, 10, or 28 days after initiation of treatment is shown. These data represent the average ± s.e.m. for 3 mice in each category. Sal, saline; Bleo, bleomycin. ^*^p < 0.05.

To begin to address whether Region I and Region II cells enter the lung from the circulation already possessing their specialized phenotype or whether they develop this phenotype in the lung, we immunolabeled PBMC from mice sacrificed 28 days after bleomycin treatment for CD45 and Pro (Figure 9A). Essentially no circulating PBMC show the CD45^high^ phenotype of Region I cells. Even the few PBMC that fall within Region II are dissimilar from the lung cells in Region II because the lung cells are almost completely in the upper half of Region II (in terms of CD45 level) while the PBMC are almost completely in the lower half (Figure 9A). These observations strongly support the notion that the fibrocyte phenotype (exemplified by high levels of CD45 on Pro+ cells) develops primarily after precursors or immature fibrocytes enter the lung. This viewpoint is further supported by preliminary studies (not shown) indicating that 28-day bleomycin PBMC never exhibit the high levels of HSP47 and CCR5 found in 28-day bleomycin lung Regions I and II.

To determine whether the accumulation of Region I cells in bleomycin lung might result from a high level of similar cells in the circulation at earlier time points, we also quantified cells in Region I + II in PBMC from saline- and bleomycin-treated mice 3, 10, and 28 days after pump implantation (Figure 9B). While there were no differences between saline and bleomycin at any individual time point, there were large differences between the time points. Just as there were similar high numbers of cells in Region II in both saline and bleomycin lung at day 3 (Figure 1), the highest level of circulating Region I + II cells was at day 3. This observation strongly suggests that these cells are mobilized from the bone marrow in response to surgery/pump implantation. After recovery from surgery, the number of circulating Region I + II cells is relatively low at days 10 and 28. This decrease at day 10 is particularly significant when saline and bleomycin data are pooled (*p* < 0.002). These observations strongly suggest either that the cells that comprise bleomycin Region I are either rapidly recruited from the circulation (and therefore never accumulate there) or that cells do not differentiate into the Region I phenotype until after they are recruited into the lung.

### Fibrocyte number and fibrosis score are highly correlated

**Figure 10:**
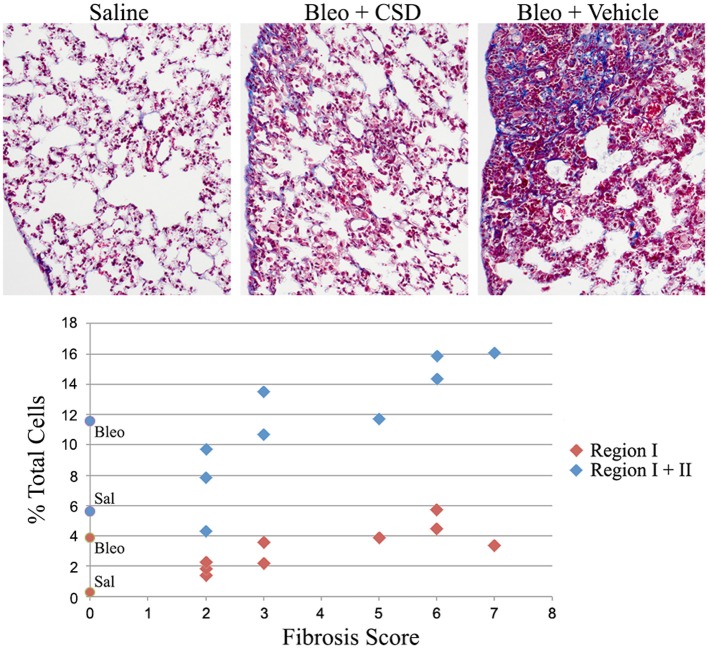
**Correlation between fibrocyte number and fibrosis score**. Mice were treated with saline or bleomycin followed 12 days later by treatment with CSD or vehicle for 2 weeks. Mice were then sacrificed on day 26 for histological evaluation and quantification of cells in Regions I and II. **Top:** Images are shown for mice treated with Saline (Fibrosis Score 0), Bleomycin + CSD (Fibrosis Score 2 to 3, 5 of 9 mice responded in this manner), and Bleomycin + Vehicle (Fibrosis Score 5 to 7). **Bottom:** For 9 mice receiving Bleomycin + CSD, their Fibrosis Score is plotted separately vs. percent of total lung cells in Region I and percent of total lung cells in Regions I + II. For reference, data from Figure 1D regarding the percent of total lung cells in Region I and Regions I + II from saline- and bleomycin-treated mice that did not receive further treatment with CSD or vehicle are presented on the y-axis as round symbols. Sal, saline; Bleo, bleomycin.

We previously demonstrated that the CSD peptide provides protection against lung fibrosis induced by the delivery of bleomycin directly into the lungs (Tourkina et al., 2008). As we have recently demonstrated that the Pump Model, which involves systemic bleomycin treatment, more closely mimics several features of SSc (Lee et al., 2014), here we have determined whether CSD also provides protection against lung fibrosis in the Pump Model. In addition, here we did not initiate CSD treatment until 12 days after the initiation of bleomycin treatment, whereas in our previous study CSD treatment began 1 day prior to bleomycin treatment (Tourkina et al., 2008). Despite the delayed treatment, five of nine mice showed noticeable improvement in tissue morphology and fibrosis score compared to mice that received bleomycin and CSD vehicle (Figure 10). When fibrosis score was plotted against fibrocyte number, a striking correlation was observed. For Region I fibrocytes, the correlation was *r* = +0.80, *p* = 0.01; for Regions I + II fibrocytes, the correlation was *r* = +0.95, *p* < 0.0001. These results strongly support the ideas that fibrocytes play an important role in the progression of lung fibrosis and that targeting fibrocytes may be an effective way to treat diseases involving lung fibrosis.

## Discussion

Here we have used flow cytometry to study the levels and immune-phenotype of fibrocytes in the fibrotic lung using a model system in which bleomycin is delivered systemically using osmotic minipumps. This Pump Model (Lee et al., 2014) produces a disease that is much more similar to human SSc than is the more common model in which bleomycin is delivered directly into the lungs. Our studies also benefited from the use of an anti-collagen I antibody that we developed against the collagen Iα1 C-terminal propeptide (referred to as Pro). The key advantage of this antibody is that the specificity of labeling can be confirmed by competition using the immunogenic peptide. Our key finding is that fibrotic lung fibrocytes can be divided into two populations: CD45^high^/Pro+ cells that we refer to as Region I and CD45+/Pro+ cells that we refer to as Region II. While Region II fibrocytes are present both in control and fibrotic lung, Region I fibrocytes are present essentially only in the fibrotic lung. The remaining cells in the lung can be classified as leucocytes that are CD45+/Pro- (Region III) and all other cells (CD45-, referred to as Region IV).

Studies on the time course of appearance of cells in Region I confirmed the association of these cells with lung fibrosis. These cells were present at the same low level as in saline-treated, control mice 3 days after the initiation of bleomycin treatment. Even at 10 days the level in the bleomycin lung was only about 30% of the level ultimately attained at day 28. Strikingly different results were attained in Region II in which a large number of cells were present 3 days after the initiation of treatment with either bleomycin or saline vehicle. This level decreased to near the baseline level at day 10 and remained near this level through day 28, although by this time the level in the bleomycin lung was slightly (and statistically significantly) higher in the bleomycin lung. Interestingly, the peak in number of cells in Region II observed in the lung at day 3 was also observed in the blood at day 3, as was the subsequent decrease. These observations strongly support the idea that the increase in cells in Region II observed in both the lung and blood at day 3 is due to the implantation of pumps and not to the content of the pumps.

While most experiments in this study were performed with 10-week old male CD1 mice, to confirm that the cells present in Regions I–III (i.e., all CD45+ cells) are bone marrow-derived, some experiments were performed with 24-week old C57/BL6 that had been irradiated, then reconstituted with bone marrow from mice expressing EGFP in all of their cells. Not only did these studies confirm that Regions I–III cells are all bone marrow-derived, they confirmed that the existence of Region I cells in fibrotic mice is not simply an unusual outcome that occurs only in CD1 mice or that occurs only in relatively young mice. It is also of interest that we observed that the level of EGFP is much higher in Regions I and II than in Region III (not shown) because the existence of GFP^high^ and GFP+ cells in fibrotic lung has recently been reported (Nakashima et al., 2013). At present, the relationship between these GFP^high^ cells and our GFP^high^ cells is a matter of speculation because our GFP^high^ cells are all Pro+ (i.e., Col I+) while the GFP^high^ cells of Nakashima et al. were reported to be <8% Col I+ (Nakashima et al., 2013).

The central findings of this study regarding the phenotype of lung fibrocytes and leucocytes are summarized in Table 2. In accord with the way that Regions I–III were defined, the median fluorescence intensities of CD45 and Pro were several-fold higher in saline control fibrocytes than in saline control leucocytes. The levels of CD45 and Pro immunolabeling were somewhat higher in fibrotic fibrocytes than in control fibrocytes, presumably because both CD45 and Pro immunolabeling were at their highest levels in Region I which is only present in the fibrotic mouse fibrocyte population. It is also noteworthy that the level of CD45 on circulating fibrocytes was much less than on lung fibrocytes either from Region I or Region II.

**Table 2:** **Phenotypic differences among fibrocytes and between fibrocytes and leucocytes**.

	**Saline fibrocytes/saline leucocytes**	**Bleomycin fibrocytes/saline fibrocytes**	**Bleomycin region I/bleomycin bleomycin region II**
CD45	2.9	1.3	2.0
Pro	5.5	1.3	1.6
CXCR4	5.4	1.7^*^	1.6^*^
CCR5	14.5	1.2	1.5^***^
HSP47	14.0	1.7^***^	1.2
CD44	28.8	0.8	2.1^*^

*In this table, Leucocytes are defined as cells in Region III and Fibrocytes are defined as cells in Region I + II. Data are the ratio of median fluorescence intensity for each pairing from Figures 1, 6–8*.

* p < 0.05,

*** *p < 0.002*.

Results with CXCR4 and CCR5 raise the interesting possibility that these two chemokine receptors have distinct roles in fibrocyte biology. While both are expressed at several-fold higher levels in saline control fibrocytes than in saline control leucocytes, the increase for CCR5 is three-fold more than for CXCR4. The levels of both CXCR4 and CCR5 immunolabeling were somewhat higher in fibrotic fibrocytes than in control fibrocytes and were at their highest levels in Region I. Interestingly, preliminary studies (not shown) suggest that the level of CXCR4 on lung fibrocytes is little more than the level on circulating fibrocytes, while the level of CCR5 on lung fibrocytes is much higher than on circulating fibrocytes. In addition, we show that the distribution on CD45/Pro scatter plots is different for the fibrocytes with the highest levels of CCR5 than for the fibrocytes with the highest levels of CXCR4, proving that these are not the same cells. Additional work needs to be done to sort out the functional significance of these observations and to determine how CXCR4 and CCR5 may differentially regulate fibrocyte biology and the role of fibrocytes in fibrosis in various organs.

The collagen chaperone HSP47 plays critical roles in the secretion, processing, and formation into fibrils of collagen I *in vitro* and in fibrosis *in vivo* (Ishida et al., 2006; Hagiwara et al., 2007). We find a huge increase in HSP47 expression in fibrocytes compared to leucocytes (Table 2). There is a further, highly significant increase in HSP47 expression in bleomycin fibrocytes compared to control fibrocytes; however, the level of HSP47 in bleomycin Region I fibrocytes is not much higher than in bleomycin Region II fibrocytes. In contrast to all the other proteins examined in this study (CD45, Pro, CXCR4, CCR5, CD44), there is a population of HSP47^high^ cells that are CD45- (i.e., in Region IV) that are present at a high level in the bleomycin lung but not in the control lung. Given that we previously observed double-positive cells that are Prosurfactant C+/HSP47+ in the fibrotic, Pump Model lung and in the lungs of SSc patients (Lee et al., 2014) and that we find that Prosurfactant C+ cells are GFP- in reconstituted mice (not shown), we propose that the many of the HSP47+ cells in Region IV will be found to be Prosurfactant C+ cells derived from type II pneumocytes.

Among the proteins examined, the greatest increase we observe between fibrocytes and leucocytes is in the expression of CD44 (Table 2). This is all the more remarkable because the level in leucocytes (Region III) is much higher still than in CD45- (Region IV) cells. This is also noteworthy because CD44 and its ligand hyaluronic acid have been reported to regulate fibrocyte differentiation (Maharjan et al., 2011), although many of the details remain to be worked out. While the level of CD44 is no higher in bleomycin fibrocytes than in saline fibrocytes, the level of CD44 is much higher in bleomycin Region I than in bleomycin Region II.

To address the importance of fibrocytes in lung fibrosis, we treated mice that received bleomycin via osmotic minipump from days 0 to 12 with CSD delivered via osmotic minipump from day 12 to 26. This treatment blocked the migration of bone marrow cells isolated from these mice toward several chemokines including CXCL12, MCP-1, MCP-3, MIP1α, and MIP1β (not shown) in an *in vitro* assay, suggesting that the recruitment of bone marrow-derived cells into affected tissues should be similarly inhibited *in vivo*. Even though CSD treatment did not begin until after fibrosis had begun to be established, this treatment also inhibited both fibrocyte number in the tissue and fibrosis score with a highly significant correlation. Together, these observations strongly support the notion that CSD blocks the progression of fibrosis by blocking the recruitment by chemokines of bone marrow-derived fibrocytes or their precursors into affected tissues.

In summary, the results of this study strongly suggest that fibrocyte differentiation occurs in large part in the target tissue, in this case the lungs. Besides highlighting fibrocyte recruitment and differentiation as potential therapeutic targets (using CSD or other agents) in the treatment of fibrotic diseases, this study also highlights CD45, CD44, CCR5, CXCR4, and HSP47 as potential therapeutic targets.

## Author contributions

Charles Reese participated in study design, performing experiments, data interpretation, and manuscript preparation. Rebecca Lee, Beth Perry, and Jonathan Heywood participated in performing experiments. Michael Bonner participated in performing experiments and in editing the manuscript. Richard M. Silver and Richard P. Visconti participated in editing the manuscript. Elena Tourkina participated in study design. Stanley Hoffman participated in study design, data interpretation, and editing the manuscript. All authors read and approved the final manuscript.

## Financial support

This work was supported by grants: USARMY/USAMRAA W81XWH-11-1-0508 (to Stanley Hoffman); NIH NIAMS R01 AR062078 and a grant from the Scleroderma Foundation (to Elena Tourkina); NIH NIAMS P60 AR049459 (Multidisciplinary Clinical Research Center) (to Richard M. Silver); and an NIH NCRR Construction Grant C06 RR015455. Elena Tourkina also received the Marta Max Award from the Scleroderma Foundation.

### Conflict of interest statement

While none of the authors have received any financial benefit from this work, Drs. Stanley Hoffman and Elena Tourkina are the Inventors on a use patent (# 8,058,227) issued to the Medical University of South Carolina on the caveolin-1 scaffolding domain peptide as a treatment for fibrotic diseases. Drs. Stanley Hoffman and Elena Tourkina are also the founders of a company, FibroTherapeutics, Inc., for the purpose of developing a drug based on the caveolin-1 scaffolding domain peptide. The authors declare that the research was conducted in the absence of any commercial or financial relationships that could be construed as a potential conflict of interest.
